# Book Review: Professional Support Beyond Initial Teacher Education: Pedagogical Discernment and the Influence of Out-of-Field Teaching Practices

**DOI:** 10.3389/fpsyg.2020.620326

**Published:** 2020-12-03

**Authors:** Qiong Zheng, Xinmin Zheng

**Affiliations:** School of Education, Shanghai International Studies University, Shanghai, China

**Keywords:** out-of-field teaching, professional development, professional learning, identity formation, initial teacher education (ITE)

The quality of education has long been a core point of attention for all stake-holders involved in education. In fact, teachers are the most important agents to guarantee the education quality (Hattie, 2009). Thus, schools and institutions strive to maintain a stable, qualified, and sustainable teaching workforce. Teacher professional development helps to better prepare teachers for diverse teaching context, enhances the well-being of teachers and improves the quality of human resources of teaching in schools. That's why it has been a global research concern (Wang and Shuttlesworth, 2020). As far as we are concerned, though acknowledged as a life-long process of learning and developing in the research of a recent decade, in real practice, the focus of teachers' professional development is still on the beginning stage. However, beyond the crucial initial stage, teachers may still face serious challenges in their career development, which either drop them out or drive them to learn being professionalized. This is the very reason why Du Plessis's book has attracted our attention. Instead of focusing on beginning teachers, Du Plessis, in her book titled *Professional Support Beyond Initial Teacher Education: Pedagogical Discernment and the Influence of Out-of-field Teaching Practices*, explores the out-of-field teaching phenomenon which is being aware of but far under-researched in previous studies (Hobbs, 2013). The book shows a great concern for teachers in challenging positions and attempts to find solutions in targeted professional support, from which all stakeholders in educational system can benefit.

The eight chapters in the book are divided into two parts. Part I lays the theoretical foundation for the whole book. Chapter One explores the capacities that teachers are typically required to possess in order to be deliver quality education, justifying the significance of a systematic and targeted professional support in filling the gap between initial teacher education (ITE) and starting a full-time position. Chapter Two elaborates on a context-conscious understanding development (C-CUD) theoretical framework, so the readers can have a tool to understand the various professional support strategies from a new perspective. The framework will help readers to link teaching to specific situation and become more conceptually and contextually aware of the issues of “how, where, why, and what.” Chapter Three investigates the education leadership and decision-making process, in which a CANNAS school leadership model (Connectedness, Awareness, Need Analysis, Negotiation, Action, and Support) is introduced. Chapter Four discusses the link between ITE and teachers' workplace learning and development.

As a whole, the remaining four chapters in Part II demonstrate the complex contexts in which educators are situated and offer a pedagogical understanding of teachers' lived experience, from which readers can perceive the variety of factors that influence teachers' pedagogical actions and professional development, such as leadership, peer support, community learning, etc. Chapter Five invites readers to enter into in teachers' real teaching world with empirical evidence, and demonstrates the necessity of targeted professional support which can help teachers to develop their capacities. Chapter Six takes a close look at the complicated teaching and learning context and analyses the influence of out-of-field phenomena on decision-making of leadership in a certain organizational culture. Chapter Seven discusses the potential influence that effective professional support can have on teachers' commitment to transformation, development of their capacities, and the building of the teacher workforce. Finally, Chapter Eight proposes several targeted recommendations for how to carry out professional support in the workplace in the future, in order to best shape a quality teaching workforce.

As the book regards initial teacher education (ITE) as the start point of teachers' identity formation, it is clear that whatever ITE a teacher undergoes initially will have an effect on them if faced with the challenge of teaching “out-of-field.” Adopting a holistic view on teachers' professional development, the book holds that teachers' identity develops consistently over time. As in this book, “out-of-field” teaching responsibilities, if required, will create a substantial gap in this identity formation process. With four philosophies as its theoretical foundation, namely situated learning, lived experience, the social constructivist learning and Hermeneutic philosophy, Du Plessis develops a theoretical framework C-CUD and provides a deeper understanding of out-of-field phenomena, which can also serve as a guide for professional support design. Previous research on teacher professional development either explores the effectiveness of ITE or investigates various strategies of professional support such as peer-mentoring, teachers' self-reflection, professional learning community, etc. Du Plessis's book, however, focuses on ITE's positive effect of preparing new teachers for their work, and further probes into the space between ITE and what goes beyond it in real workplace, which closes the gap between teachers' learning in ITE and teachers' workplace learning when facing “out-of-field,” and thus well bridges the previous two kinds of research.

In addition to the theoretical contributions discussed above, the book offers a clear theoretical lens into professional support when “out-of-field” teachers are the target participant group under investigation. Du Plessis has followed the line of conceptualization of professional support (Knapp, 2003) and argued that professional development should be specific, context-based, and continuous at the institutional level, while professional learning should be collegial-supported, inquiry-encouraged, and life-long at the level of individual teachers. The book regards professional support as a strategy that can facilitate teachers' learning and development, and thus provides a more nuanced understanding in differentiating the type of support at the institutional level from that at the individual level.

If the book were to be considered for future edition, we would put forward the following suggestions. First, the book would be more thorough if it could offer a more comprehensive solution, preferably a model, for policy makers and educators to the challenges faced by the out-of-field teachers. Second, the book apparently investigates the out-of-field teachers' professional development through the lens of an out-sider, and it would be more convincing if it could explore deeper into the inner world of the out-of-field teachers from their own perspective. Third, the book is strong in its theoretical foundation, however, it would be fuller if it could include more data collected in front-line classrooms when illustrating specific theories.

Overall, Du Plessis' book is an important contribution to research on out-of-field education. Its rich empirical data well describes the impact of this comparatively unexplored issue on education quality. The book further provides a theoretical framework for teacher professional development that can be referred to by both leadership and individuals, and also offers practical suggestions for all stakeholders in education. The book is reader-friendly, easy to understand, informative, and well-organized. As researchers in the field of teacher professional development, we would highly recommend it to teachers, school leaders, ITE developers, education authorities, researchers, doctoral students, and anyone who is interested in the related topic.

## Author Contributions

QZ has written the book review. XZ helped to examine and modify the review.

## Conflict of Interest

The authors declare that the research was conducted in the absence of any commercial or financial relationships that could be construed as a potential conflict of interest.

## References

[B1] Hattie J. (2009). Visible Learning: A Synthesis of Over 800 Meta-Analyses Relating to Achievement. London: Routledge.

[B2] Hobbs L. (2013). Teaching ‘out-of-field’ as a boundary-crossing event: factors shaping teacher identity. Int. J. Sci. Math. Educ. 11, 271–297. 10.1007/s10763-012-9333-4

[B3] Knapp M. (2003). Professional development as a policy pathway. Rev. Res. Educ. 27, 109–131. 10.3102/0091732X027001109

[B4] Wang Y. Shuttlesworth D. (2020). Close the achievement gap with professional development. Int. J. Teach. Educ. Profess. Dev. 3, 88–101. 10.4018/IJTEPD.2020010106

